# Seeking Correlation Among Porin Permeabilities and Minimum Inhibitory Concentrations Through Machine Learning: A Promising Route to the Essential Molecular Descriptors

**DOI:** 10.3390/molecules30061224

**Published:** 2025-03-09

**Authors:** Sara Boi, Silvia Puxeddu, Ilenia Delogu, Domenica Farci, Dario Piano, Aldo Manzin, Matteo Ceccarelli, Fabrizio Angius, Mariano Andrea Scorciapino, Stefan Milenkovic

**Affiliations:** 1 Department of Chemical and Geological Sciences, University of Cagliari, S.P. 8 km 0,700, I-09042 Monserrato, CA, Italy; sara.boi95@unica.it; 2 Department of Biomedical Sciences, University of Cagliari, S.P. 8 km 0,700, I-09042 Monserrato, CA, Italy; silvia.px@live.com (S.P.); deloguilenia@gmail.com (I.D.); aldomanzin@unica.it (A.M.); 3 Department of Life and Environmental Sciences, University of Cagliari, S.P. 8 km 0,700, I-09042 Monserrato, CA, Italy; domenica.farci@unica.it (D.F.); dario.piano@unica.it (D.P.); 4 Department of Physics, University of Cagliari, S.P. 8 km 0,700, I-09042 Monserrato, CA, Italy; matteo.ceccarelli@dsf.unica.it (M.C.); stefan.milenkovic@dsf.unica.it (S.M.)

**Keywords:** antibiotics, drug design, artificial intelligence, infectious diseases, in silico screening, liposomes, molecular dynamics

## Abstract

Developing effective antibiotics against Gram-negative bacteria remains challenging due to their protective outer membrane. With this study, we investigated the relationship between antibiotic permeation through the OmpF porin of *Escherichia coli* and antimicrobial efficacy. We measured the relative permeability coefficients (RPCs) through the bacterial porin by liposome swelling assays, including non-antibacterial molecules, and the minimum inhibitory concentrations (MICs) against *E. coli*. We developed a machine learning (ML) approach by combining classification and regression models to correlate these data sets. Our strategy allowed us to quantify the negative correlation between RPC and MIC values, clearly indicating that increased permeability through OmpF generally leads to improved antimicrobial activity. Moreover, the correlation was remarkable only for compounds with significant permeability coefficients. Conversely, when permeation ability is low, other factors play the most significant role in antimicrobial potency. Importantly, the proposed ML-based approach was set by exploiting the available seminal information from previous investigations in order to keep the number of molecular descriptors to the minimum for greater interpretability. This provided valuable insights into the complex interplay between different molecular properties in defining the overall outer membrane permeation and, consequently, the antimicrobial efficacy. From a practical perspective, the presented approach does not aim at identifying the “golden rule” for boosting antibiotic potency. The automated protocol presented here could be used to inspect, in silico, many alternatives of a given molecular structure, with the output being the list of the best candidates to be then synthesized and tested. This could be a valuable in silico tool for researchers in both academia and industry to rapidly evaluate novel potential compounds and reduce costs and time during the early drug discovery stage.

## 1. Introduction

**Figure 1:**
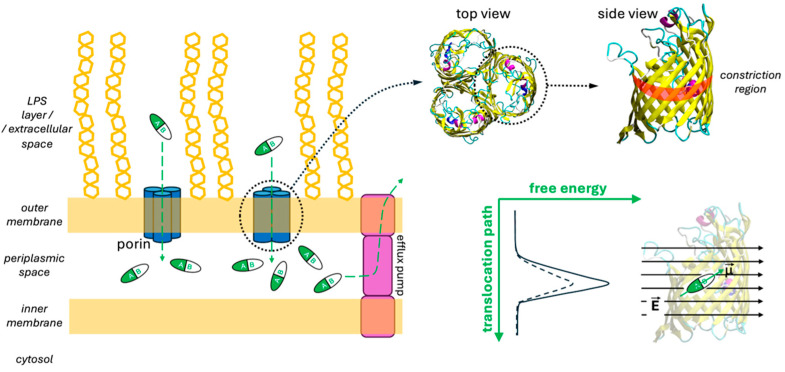
Porins are water-filled transmembrane trimeric protein channels localized in the outer membrane of Gram-negative bacteria. These are the main entry path for most of the known hydrophilic antibiotics. However, the accumulation of the latter in the periplasmic space is limited by efflux pumps (besides the possible presence of degradative enzymes). Each of the three identical porin’s monomers has a beta-barrel structure, characterized by an hourglass-shaped lumen. The constriction region is located roughly halfway along the antibiotic translocation path, representing the main energetic barrier. This is essentially steric, but it can be partially attenuated by the proper alignment of the antibiotic’s electric dipole to the porin’s intrinsic electric field.

The development of antibiotics against Gram-negative bacteria has proven more challenging compared to other microorganisms, owing to the presence of their outer membrane (OM; Figure 1) that effectively protects the bacterial cell from toxic substances [1,2,3,4]. Porins, water-filled protein channels within the OM (Figure 1), control the permeation of small water-soluble molecules [5], including most currently available antibiotics and inhibitors. Not surprisingly, porin mutations or their altered expression are found in numerous drug-resistant strains [3,6]. Regardless of a drug’s potency against its isolated target, efficacy will be low if it fails to reach the latter inside the cell. Thus, drug permeation through the OM is crucial for its overall effectiveness. However, permeation is often overlooked in conventional antibiotic development campaigns. Gram-negative bacteria also exploit efflux systems to resist antibiotics (Figure 1), but permeation through the OM remains of uttermost importance for the drug to reach proper accumulation inside the bacterial cell to exert its activity [7]. Aiming at novel effective antibiotics against Gram-negative pathogens, an understanding of the molecular basis behind penetration through the porins is crucial [4,8,9]. *Escherichia coli* is one of the most studied and well-known Gram-negative bacteria, and it is often used as a model organism. The two main porins of *E. coli*, OmpF [10] and OmpC [11], were among the first outer membrane proteins (Omps) whose 3D structures were solved at high resolution. OmpF and OmpC are homotrimers made up of three identical beta-barrel monomers (Figure 1). The longest loop (L3) in each monomer folds back into the pore, giving it an hourglass shape (Figure 1). This constriction region (CR) acts as the main steric barrier limiting the diffusion of molecules (and ions) through the channel. For a long time, the slightly smaller pore size and lower permeability of OmpC compared to OmpF led to the belief that molecular size was the primary factor determining their permeability to drugs, with their relative expression dictated by the environment’s conditions [12,13,14]. However, despite extensive research, both experimental and theoretical, definitive rules for designing new drugs that can effectively cross the OM remain elusive. The focus of these studies has typically been the CR, with various types of interactions between the porin and the antibiotic, such as hydrogen bonds, hydrophobic contacts, and salt bridges, being proposed to explain the differences in permeability observed for different molecules. The idea of a specific binding or affinity site within the channel has even been proposed in the literature as a potentially simple model to describe the clearly complex and multivariate process of drug permeation. The available evidence strongly suggests that drug permeability depends on general physicochemical properties, such as size, net charge, charge distribution, conformational flexibility, and hydrophobicity. However, no clear, robust correlation has been found between permeation rates and any of these individual characteristics, also due to the lack of a reliable method to accurately measure permeability [15].

Single-molecule cell-free electrophysiology experiments have highlighted the crucial role of electrostatic interactions between polar molecules and these protein channels [16]. Numerous theoretical studies have attempted to characterize the electrostatics within the porin channels, from Karshikoff’s work using the Poisson–Boltzmann equation [17] to the molecular dynamics simulations of Im and Roux describing a screw-like electrostatic topology in OmpF [18]. More recently, some authors proposed an all-atom simulation method to calculate macroscopic electric fields in nanometric water-filled channels by exploiting water polarization density, allowing comparison across systems and conditions [19]. Then, OmpF was used as a model pore, with norfloxacin serving as a molecular probe to quantify this internal electric field in bacterial porins [20]. The findings bolstered that molecular transport is strongly influenced by the molecule’s ability to conform to the pore’s electrostatic landscape, with almost negligible dependence on the presence of lipopolysaccharides (LPS) [21]. The following general picture emerged: while molecules experience a significant decrease in conformational entropy upon entering the CR, the free energy increase can be offset by the favorable alignment of the molecular electric dipole with the channel’s internal electric field (Figure 1). Moreover, a “pre-orientation” region above the CR, as found in OmpF, facilitates translocation, whereas weak or oppositely directed fields, as observed in OmpC or two resistant mutants [22], can hinder molecular passage by imposing unfavorable orientations at the CR’s entrance.

Building on these insights, a predictive function has been developed and published, which can estimate the permeability coefficient of a molecule through the *Enterobacteriaceae*’s general porins based on parameters such as size, net charge, and electric dipole [23]. Such a scoring function, developed for permeability prediction, was successfully extended to predict accumulation in intact bacteria without parameter retraining, demonstrating its ability to discriminate different accumulation levels [24]. However, it was parameterized on the experimental relative permeability of a few antibiotics (nine in total), all of which were β-lactams. Moreover, it was inherently based on the mathematical formulation of several energy interaction terms between the substrate and the specific porin’s steric and electrostatic profile.

Following a different approach, seeking more generality and flexibility, some authors have recently shown how reliable predictive models can be obtained by using machine learning (ML) with a limited number of interpretable descriptors related to shape, size, and electrostatics [25], using a rather large accumulated data set from the literature [7]. Furthermore, it has been shown how improved performance can be achieved by incorporating statistical descriptors from MD simulations and electric dipole moment data, bolstering previous observations.

**Figure 2:**
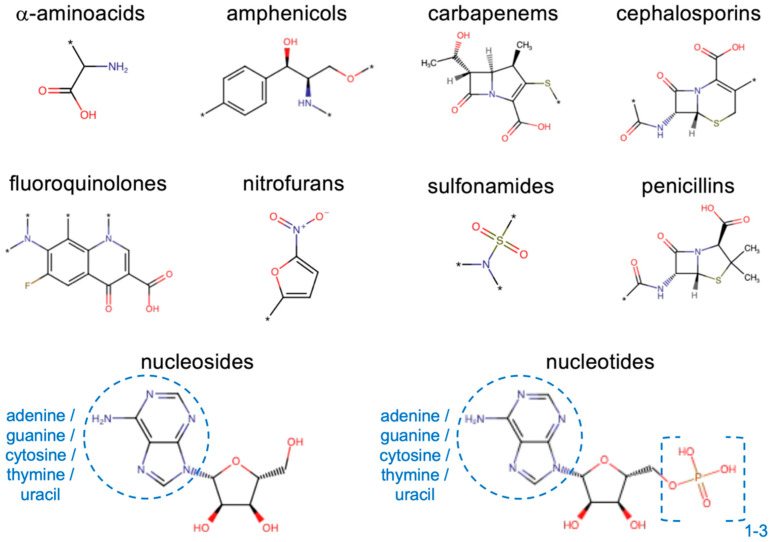
Main molecular classes investigated. The asterisk represents a generic atom or chemical group.

In this work, we wanted to test the same strategy to generate predictive models for permeability through porins, taking OmpF from *E. coli* as a prototypical example. Experimental measurements of relative porin permeability were extended to a set of 30 compounds from different molecular classes. In addition, we also took advantage of data from the literature [23,26], up to a total of 41 compounds (Figure 2). These data were used to build a classification model with ML. Then, a regression model was developed on that basis, able to predict continuous values of relative permeability for unknown compounds, by starting just from their SMILES string with a fully automatic pipeline. By including both antibiotics and non-antibiotics in the data set, in fact, we did not want to focus on known antibacterials, but we aimed at extending the approach on a wider chemical space, so as to help the discovery of good permeants belonging to novel molecular classes to be then developed as antibiotics or as potential vectors for poorly permeating antibiotics. Finally, we exploited minimum inhibitory concentration (MIC) measurements performed on *E. coli* for a set of 38 antibiotics from different classes (Figure 2) to quantify the correlation with through-porin permeation.

## 2. Results and Discussion

### 2.1. Liposome Swelling Assays

**Figure 3:**
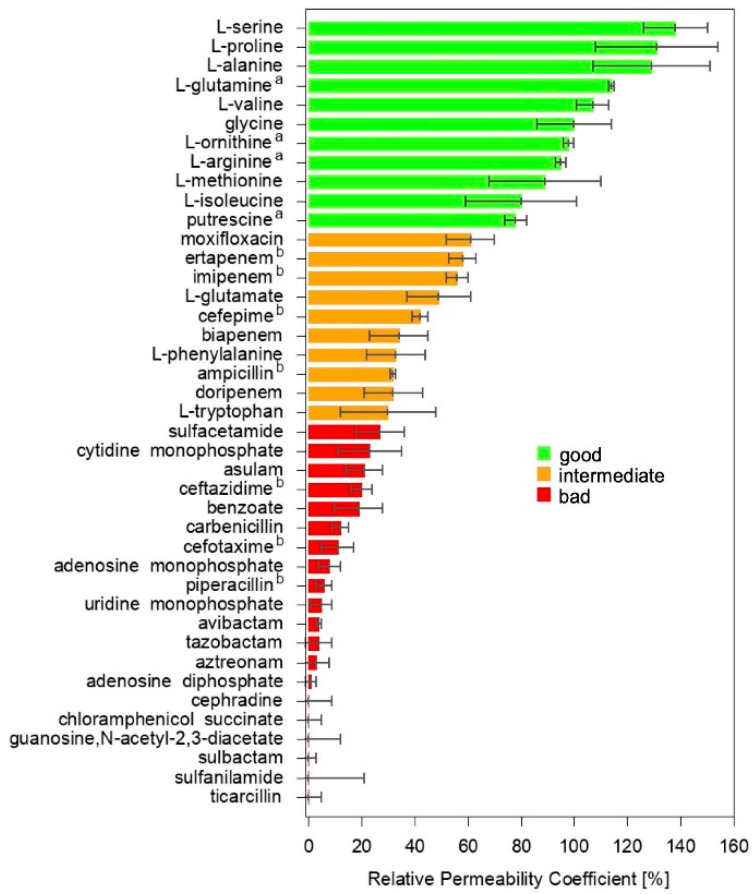
The relative permeability coefficient (RPC) of the 41 selected substrates through OmpF from *E. coli* is shown (glycine is the 100% reference). Data for the compounds labelled with a and b have been taken from ref. [26] and ref. [23], respectively.

The relative permeability of OmpF from *E. coli* to a set of 30 substrates was estimated with the liposome swelling assay (LSA), and an additional 11 data points were taken from the literature (same method) [23,26]. The data set, 41 substrates in total, comprised both antibiotics and non-antibiotic substances (Figure S1). They were selected to sample different chemical classes, namely, amphenicols, amino acids, b-lactamase inhibitors (including b-lactams and diazabicyclo compounds), carbapenems, carbohydrates, cephalosporins, fluoroquinolones, monobactams, nucleosides, nucleotides, penicillins, and sulfonamides. Besides having a sufficient water solubility to be investigated with LSA (logP at pH 7.4 was in the range between −0.1 and −14.9), these molecules were selected to sample a wide range of values for different chemico-physical descriptors considered to have significant impact on translocation through general porins [6,7,9,13,14,16,20,22,24,27]; namely, the molecular weight (75–546 g/mol) and Van der Waals volume (68–435 Å^3^), rotatable bonds (1–11) and ring counts (0–5), Van der Waals area (120–691 Å^2^), polar surface area (40–258 Å^2^), minimal (19–80 Å^2^) and maximal (25–120 Å^2^) projection area, and the electric net charge (−2.5–+2.0/e) and dipole moment (0.0–66.2 D). The values obtained for the minimum energy conformer of the main protonation state at pH 7.4, as evaluated through MarvinSketch, are listed in Table S1. Figure 3 shows the relative permeability coefficient (RPC, relative to glycine) obtained for all the substrates in the data set.

Amino acids are the best permeators, while most of the antibiotics exhibited a lower RPC. Nucleosides and nucleotides are the worst permeators. Although a rough correlation with the molecular size can be identified at a first glance, also bolstering previous observations [28], many discrepancies are clear. For example, a number of amino acids showed a comparable, or even superior, permeation with respect to glycine, despite their larger volume. Methionine and glutamate have a comparable size but the former showed a much faster permeation than the latter, bolstering the notion that the charge does have a role [23]. Several antibiotics, such as moxifloxacin, ertapenem, and imipenem, exhibited better permeation than phenylalanine or tryptophan, for instance, in spite of their much larger size.

In fact, when the correlation between each of the above-mentioned chemico-physical descriptors and the RPC was calculated (Table S3), a mild negative correlation was found for all the size-related descriptors. This result was expected in the light of the hourglass shape of the OmpF lumen, in agreement with the literature [12,13,14]. However, the only moderate correlation clearly indicated the additional importance of other descriptors, leading to the above-noted discrepancies in the RPC order. The net charge was found to have a mild positive correlation with the RPC, in agreement with some previous reports [7,29] and the net negative charge of the protein channel [22]. Poor correlation was found for rotatable bonds, the number of fused rings, and the electric dipole moment. The moderate negative correlation of the ring count is probably due to the positive correlation between the latter and the size of the substrate. Finally, no correlation was found with the logP, as expected for hydrophilic substrates whose penetration mechanism involves passage through water-filled pores.

The permeation of these small molecules through general porins is a multivariate process, such that simple univariate correlations simply fail in providing clear rules for drug design. Moreover, the very general and simplistic rules of reducing the size and adding positively charged groups do not appear to be widely applicable to increasing the permeability coefficient of existing antibiotics (or to discovering new scaffolds), since their core structure must obey to specific constraints dictated by the geometry and the chemistry of the active site on the target. By dividing, quite arbitrarily, the 41 substrates into good, intermediate, and bad permeators (Figure 3), the distribution of the selected descriptors was calculated and is reported in Figure S3. No clear distinction is offered by any of the widely employed descriptors. The good permeators appeared to be characterized by a smaller size, but it is worth remarking that these were mostly amino acids. In fact, a wide overlap was obtained with the distribution of all the three categories for all the descriptors. Among the size-related ones, the minimal projection area appeared to be a little more discriminant, suggesting that the size per-se is not as critical as the effective molecular cross-section during the translocation [22,30] and indicating that conformational plasticity should be important. However, descriptors such as the mere number of rotatable bonds and rings, widely used as indicators of conformational plasticity and structure rigidity, respectively, did not represent effective classification parameters. Their positive correlation with molecular size must be considered, due to the fact that the larger the size, the higher the number of rotatable bonds and/or rings that can be accommodated. Moreover, the number of rotatable bonds, per-se, does not provide any information on whether that specific molecule is prone to change conformation (e.g., in the presence of bulky substituents) or about its impact on the overall molecular configuration (e.g., small terminal groups). In fact, an important flaw of correlating RPC with molecular descriptors is represented by the inherent static nature of the latter when they are evaluated by typical chemoinformatics software on one single conformer. Many molecular dynamics simulation studies have shown the importance of the substrate conformational plasticity along its route through the porin. The value of many chemico-physical descriptors can have wide fluctuations as a function of the conformational changes. Consequently, their mutual correlation and their correlation with the porin permeability can also be much different from what was obtained from the simple correlation analysis discussed so far. As far as the charge is concerned, its net value cannot distinguish, for instance, one non-ionic from one zwitterionic substrate, or one mono- from one poly-ionic molecule sharing the same net charge. Charge distribution must be considered. The simplest descriptor is the dipole moment, whose value can have wide fluctuations as a function of the conformational changes.

### 2.2. Minimum Inhibitory Concentrations

**Figure 4:**
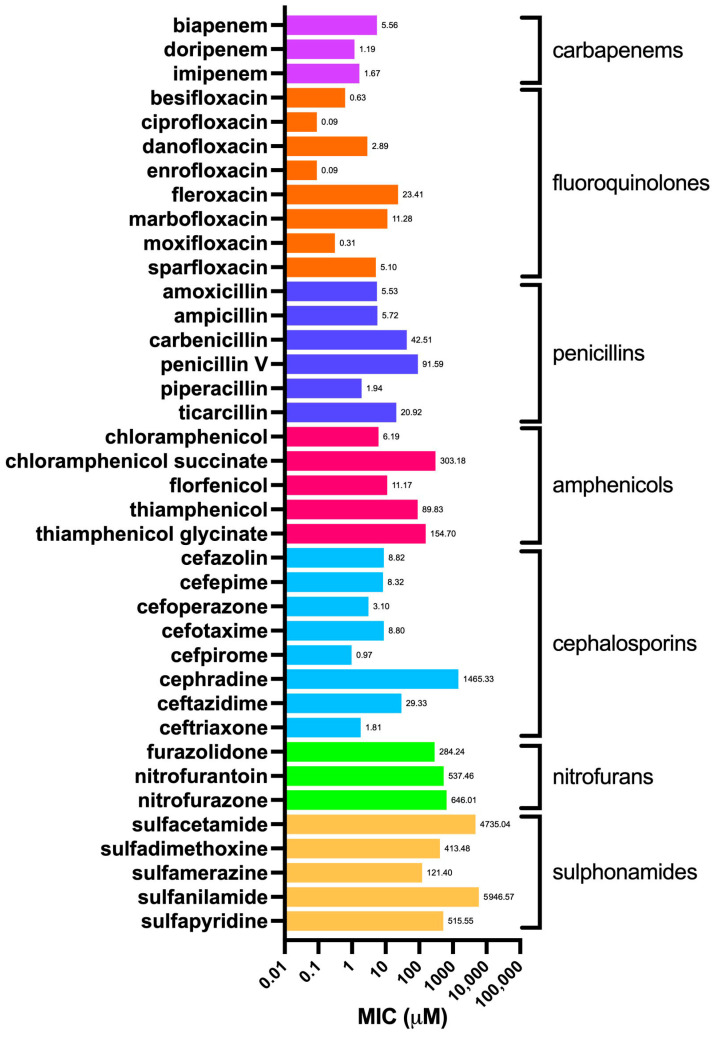
Minimum inhibitory concentration (MIC) values against *E. coli* of the selected antibiotics (Figure S2), grouped by class.

**Figure 5:**
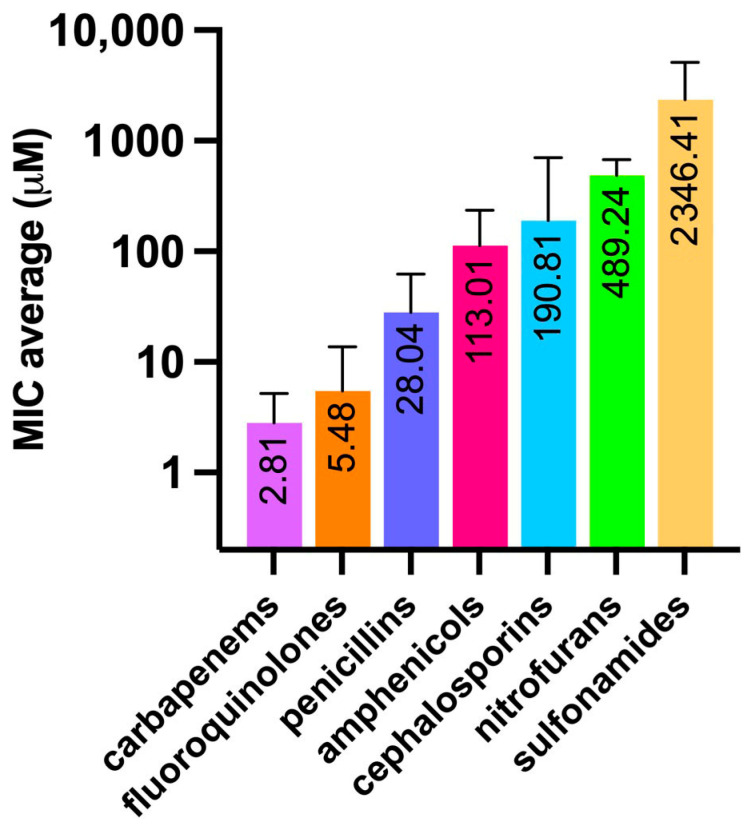
Average MIC values (µM), among different antibiotic classes, obtained against *E. coli*. Bars indicate the standard deviation.

In order to determine the MIC values, as a biological model we used the same *E. coli* strain used for OmpF production, which was then purified and employed in the proteoliposome preparation process. We challenged *E. coli* with 38 selected antibiotics, each exerting their effect by different mechanism of action and belonging to different classes; namely, cephalosporins, penicillins, carbapenems, fluoroquinolones, amphenicols, sulfonamides, and nitrofurans (Figure S2). The corresponding values of the chemico-physical descriptors evaluated through MarvinSketch are listed in Table S2. Figure 4 shows the MIC of all tested drugs, grouped by class; Figure 5 shows the average value for each class.

The obtained values were in general agreement with those reported for *E. coli* as per EUCAST [Testing TECoAS], although with some grade of variability as the latter are based on different *E. coli* strains as well as on clinical isolates. As expected, the most dated antibiotics, such as sulfonamides and nitrofurans, showed the highest average MICs. In particular, sulfacetamide and sulfanilamide showed the highest MICs (4735 and 5947 µM, respectively) among all the antibiotics tested (Figure 4). In addition, the difference between the first- and fourth-generation cephalosporins was remarkable, such as in the case of cefradine vs. cefpirome, with MICs of 1465 and 0.97 µM, respectively. This observation is primarily justified by the narrow-spectrum activity against Gram-positives of the former, compared to the broader-spectrum of the latter, which is highly effective also against Gram-negatives and which is endowed with increased resistance to beta-lactamase enzymes [31]. Penicillins show a similar trend when the aminopenicillins (i.e., amoxicillin and ampicillin), designed to affect Gram-negatives, are compared to the natural penicillins, such as penicillin V (also known as phenoxymethylpenicillin), which, together with penicillin G (benzylpenicillin), exerts a potent effect mainly against Gram-positive bacteria, those belonging to the *Staphylococcus* genus in particular [32]. Amphenicols, fluoroquinolones, and carbapenems differ from this pattern, showing lower and more consistent MIC values within their respective classes. This is likely due to their broader spectrum of action and relatively more recent discovery compared to “first-generation” antibiotics, such as sulfonamides, penicillins, and cephalosporins.

### 2.3. Machine Learning to Correlate MIC to RPC

**Figure 6:**
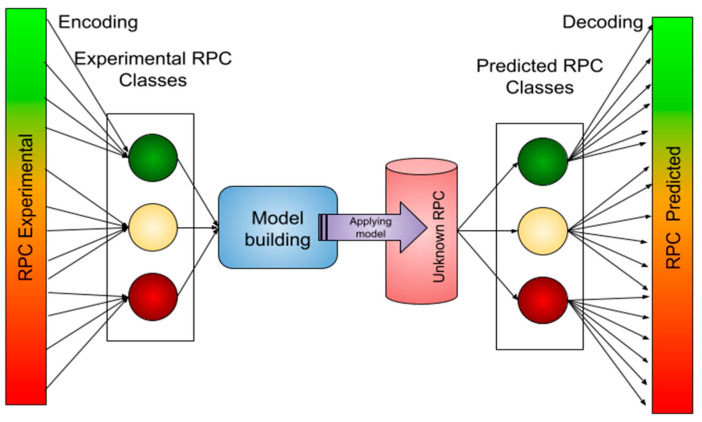
Schematic overview of the modeling procedure performed with the given data by ML techniques.

The availability of relatively few RPC data largely limits the estimation of MIC dependence on permeability. Here, we present an approach to first encode our RPC data into three classes and then create a classification model that is applied to molecules with a known MIC but unknown RPC. Once the prediction is made, the predicted classes are decoded into continuous RPC values by implementing the weighted probability function described in the SI. The procedure is depicted in Figure 6.

Training features are selected in order to combine the static molecular descriptors obtained from Marvin and the ones obtained from MD simulations (see the Methods), with a total number of 39 features. In fact, the latter are statistical descriptors (mean, standard deviation, skewness, and kurtosis) of defined molecular descriptors (minimum fitting ellipsoid axes and the dipole moments, i.e., transversal, longitudinal, and total), as summarized in Table S4. The only exception is the net charge of the simulated molecule, which must be an integer, unlike the one given by Marvin.

The first step is classification. Starting from the initial guess given in Figure 3, we tested different ML models with variable classes’ boundaries. Eventually, the optimum distribution for encoding was BAD (0–32%), INTERMEDIATE (33–62%), and GOOD (≥63%), our models giving a uniform performance as shown in Table 1. Furthermore, we decoded our classification by means of weighted probabilities as explained in SI to obtain the RMSE for each of the trained models. The best performing model was Logistic Regression with ElasticNet regularization (LR-III). With a RMSE of 22.6%_RPC_, it was the closest to the experimental average standard deviation of 8.2%_RPC_. Therefore, this was our model of choice.

**Figure 7:**
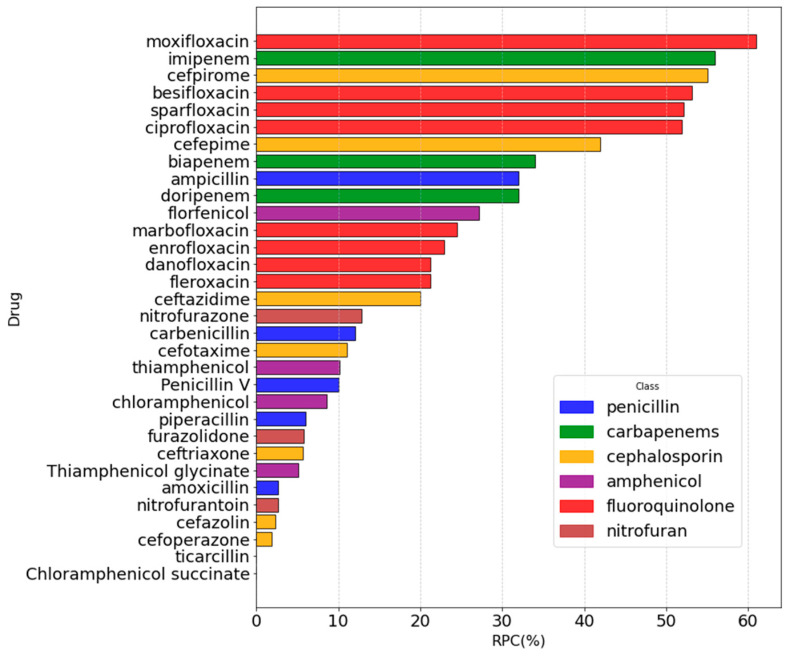
Summary of both the experimental and predicted (LR-III) RPC values for all the studied antibiotics. Colors were assigned according to the antibiotic class.

We applied the selected model, LR-III, to the antibiotics with known MIC values but devoid of experimental RPC, again performing the classification and then decoding to continuous RPC values. As a result, we have assigned RPC values either experimentally or computationally to all the antibiotics with known MIC values (Figure S2). Figure 7 shows the results, where we have illustrated the RPC distribution over the antibiotics and corresponding classes.

It is worth noting that ML predictions did not fall outside of the existing range and did not overestimate RPC values, aligning very well with the available experiments. Moreover, the LR-III model did not overestimate class members. Finally, we could investigate the relationship between the MIC and the permeability coefficient through the OmpF porin. The logMIC values were reported as a function of the decoded RPCs from each of the models. Then, a linear function was fitted to the data points. The fitting parameters are given in Table 2.

**Figure 8:**
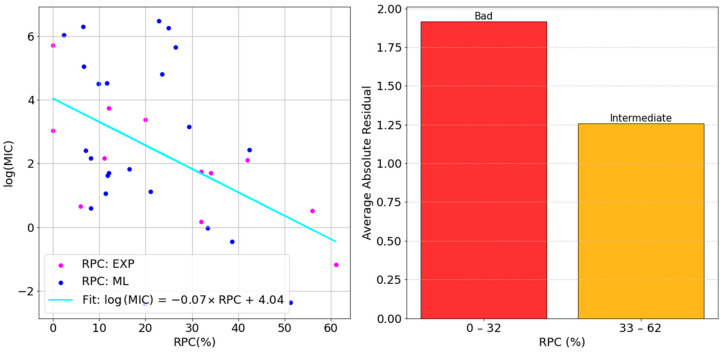
Correlation analysis of the MIC vs. RPC data points through the Logistic Regression model with ElasticNet regularization (**left**) and the residual analysis (**right**).

For all our models, a comparably negative slope was obtained. Although the most negative one was found for XG-III, we continued focusing on the best-performing model, LG-III. This case is shown in Figure 8.

The general trend of our predicted permeability values followed the experimental one very well, bolstering the validity of the presented approach. The largest deviations from the straight line (i.e., largest variability) were found on the low RPC side, while the average absolute residual decreased when increasing the permeability coefficient. This suggests that, when permeability through OmpF is rather low, other factors might be predominant for the MIC value. The latter is the outcome of multiple factors, in fact, including membrane permeability, but also the activity on the target, the specific mechanism of action, the localization of the target (whether in the periplasm or in the cytosol), and the susceptibility to degradative enzymes and efflux pump systems. All of these significantly contribute to the overall activity and can potentially diminish, or even cancel, the weight of porin permeability. The activity on the isolated target is of uttermost importance. It would be useless improving the permeability of inactive compounds. However, improving that of a moderately active molecule could make a difference with respect to another very active compound which is unable to cross the outer membrane. Additionally, the localization of the target within the cell should be considered in detailed studies. In this study, we focused on porin permeability, OmpF from *E. coli* in particular, but beside other porins, inner-membrane permeation is also important for the antibiotics with a cytosolic target. Moreover, while antibiotic accumulation in the periplasmic space is increased by good porin permeation, which is crucial to have high activity, both degradative enzymes and efflux pumps decrease it by ruining the active core of the antibiotic structure or scavenging and pushing it back to the extracellular space, respectively. The present model is intended as an example of a more widely applicable strategy. Other porins shall be included in the design and, in the future, additional homogenous data sets about the above-mentioned factors shall be incorporated to better understand the large deviation obtained for certain molecules with low RPC.

Our results showed that in the low RPC regime, the MIC can be either very large or not, and it might not necessarily improve if the RPC is improved. On the other hand, when the permeability coefficient through OmpF is larger, a clear anti-correlation with the MIC was found. This indicates that permeation efficacy assumes an increasingly important role among the different factors affecting antimicrobial potency. These results showed that, in the intermediate RPC regime, by increasing the permeability coefficient of a given antibiotic, its activity will increase. From a practical perspective, the presented approach does not aim at identifying strict golden rules for permeation improvement. One might be tempted to identify distinct structural features by comparing the Lewis structures of molecules that exhibit both a high RPC and low MIC and then incorporate these features into the structure of a given compound, expecting an improvement in antimicrobial activity. However, this approach would oversimplify the problem and represent a step back towards the previous attempts documented in the literature. Besides the limited size of the current data set, a single descriptor (or very few descriptors) cannot solely account for antimicrobial activity. Instead, a suitable combination of descriptors is required, which is where ML plays a crucial role.

Our ML model, which is expected to improve further with the inclusion of additional data, considers a smaller number of physicochemical molecular descriptors compared to conventional approaches. Importantly, it incorporates their variability, reflecting structural plasticity. Special focus is given to two key features: the distribution of atoms (modeled by the minimum fit ellipsoid) and the distribution of electric charge (characterized by the dipole moment). These two property-based descriptors provide a robust foundation that can be conveniently enriched with additional data or high-quality molecular descriptors while preserving high model’s interpretability.

The contribution of statistical descriptors is detailed in the SI. Figure S4 presents insightful distributions of the variables used in ML training. The size distribution plays a decisive role in distinguishing good permeators, with strict boundaries observed. However, the differentiation between intermediate and poor permeators is more subtle and complex, though some key patterns emerged. Intermediate permeators tend to exhibit a negative skewness in the middle ellipsoid axis (ELB_skew), suggesting their ability to contract in response to external conditions. A similar trend is observed for the transversal dipole skewness (d_T_skew). However, this must be considered in conjunction with the transversal dipole mean value (d_T_mean), indicating that intermediate permeators sample values exceeding 15 Debye more frequently. Moreover, both good and intermediate permeators exhibit lower standard deviations of the transversal dipole (d_T_std_eff) compared to poor permeators, suggesting the need for low fluctuations of the dipole during the translocation. In general, the importance of the different molecular features must be assessed mathematically, directly from the selected model, as demonstrated in Table S5. The latter highlights that the statistical effect of the transversal dipole moment is significant, in agreement with the evidence collected so far, with three out of four statistical moments ranking among the top 10 most important features.

Together, these factors contribute to a relatively simple yet interpretable model that also proves to be highly effective. The automated and interpretable protocol presented here can be applied to explore a wide range of hypothetical variations of a given molecular structure. By systematically evaluating numerous possible sidechains, substituents, isomers, and other modifications, the pipeline generates a shortlist of promising candidates, which can then be synthesized and experimentally tested. Of course, the availability of molecular synthesis methods is crucial. Our ML-based protocol begins with the SMILES string representing the entity for which the RPC is to be predicted. For large-scale screening, a predefined list of SMILES strings must be provided. The user must decide whether to focus the search on a specific class of compounds or a broader chemical space and whether to initially filter out ‘exotic’ compounds or to explore synthesis methods for the top in silico screening results later.

## 3. Materials and Methods

### 3.1. Drugs and Substrates

All drugs and substrates were purchased from MedChemExpress (Monmouth Junction, NJ, USA) with minimum purity of 95%, and used without further purification. Amino acids and carbohydrates were purchased from Merck (Darmstadt, Germany) with the same purity, as like the buffers and all the other chemicals. Lipids were purchased from Avanti Polar Lipids (Alabaster, AL, USA).

### 3.2. Protein Expression and Purification

*Escherichia coli* BL21 Gold (DE3) Omp8 cells [33] were transformed with the plasmid pGOmpF harboring the *ompF* gene of *E. coli* K12 and grown at 37 °C for 24 h under agitation (200 rpm). Cells were harvested by centrifugation at 5000× *g* for 10 min at 4 °C, resuspended in lysis buffer (20 mM Tris pH 9.0, 2.5 mM MgCl_2_ · 6H_2_O, 0.1 mM CaCl_2_ · 2H_2_O), treated with 100 U DNase I (DNase I recombinant, RNase-free, Roche CustomBiotech, Mannheim, Germany), and eventually disrupted using a French pressure cell (3× 1100 psi). Subsequently, unbroken cells were removed by low-speed centrifugation (2× 5000× *g* for 15 min at 4 °C) and cell envelopes were collected by high-speed centrifugation (48,000× *g* for 15 min at 4 °C). The cell envelope pellet was then resuspended in Buffer A (100 mM sodium phosphate, pH 7.4), homogenized, and subjected to a two-step solubilization: the first at 0.14% (final concentration) of n-dodecyl-β-D-maltoside (β-DDM) for 30 min at 25 °C, followed by centrifugation (48,000× *g* for 15 min at 4 °C); the obtained pellet was then resuspended in Buffer A, homogenized, solubilized at 1.4% β-DDM (final concentration) for 30 min at 25 °C, and finally centrifuged (48,000× *g* for 15 min at 4 °C). The supernatant was diluted in a ratio of 1:20 with Buffer A and concentrated using a Vivaspin 20 ultrafiltration membrane (Sartorius, Göttingen, Germany) with a cutoff of 100 kDa to a volume of ~200 μL. The concentrated sample was loaded on a Size Exclusion Chromatography (SEC) Superdex 200 (GE Healthcare, Chicago, IL, USA) previously equilibrated with Buffer B (100 mM sodium phosphate, pH 7.4, 0.05% β-DDM) at a flow rate of 0.5 mL/min. The main peak obtained in this step was used for functional assays. The chromatography columns were subjected to the ReGenFix procedure (https://www.regenfix.eu/, accessed on 6 March 2025) for regeneration and calibration prior to each use [34].

### 3.3. Vesicle Preparation

Liposome stock solutions were prepared as previously described [20] by mixing weighted amounts of *E. coli* total lipid extract in 1 mL of CHCl_3_/CH_3_OH 1/1 *v*/*v*. The organic solvent was evaporated under a gentle nitrogen stream to form a dried film at the bottom of the vial and then evacuated overnight. Then, the dried film was resuspended with 400 µL of properly diluted OmpF stock solution, or with a solution of the corresponding protein stabilizing detergent only, for the proteoliposome and the control liposome preparation, respectively. The solution was vortexed, sonicated for 10 min, dried in a water bath at 40 °C by gentle vacuum pumping through an anhydrous sodium sulfate trap for 30 min, and then transferred to a desiccator overnight. The next day, the dried film was resuspended with about 1 mL of 23 mM HEPES buffer (4-(2-hydroxyethyl)-1-piperazineethanesulfonic acid) at pH 7.4 in order to have 11 mg/mL and 10 μg/mL of lipid and protein final concentration, respectively. The buffer was also supplemented with dextran (average molecular weight ~40 kDa). The osmolarity of resuspending solutions was 35 + 14 mOsm/L, due to the buffer and dextran, respectively. All solutions were passed through a 0.2 μm filter before use to remove dust that could possibly interfere with the successive dynamic light scattering (DLS) and optical density (OD) measurements. Osmolarity measurements were performed with the osmometer OsmoSpecial-1 (Astori Tecnica, Poncarale (BS), Italy). Finally, vesicle solutions were vortexed for 1 min every 10 min for 1 h and then treated with 5 freeze–thaw cycles with liquid nitrogen and a 40 °C water bath, respectively. DLS measurements were performed to assess vesicle size distribution with the Zetasizer Nano ZS (Malvern Pananalytical, Malvern, UK). An aliquot of 60 μL of the vesicle stock solution was pipetted into 1 mL of an isotonic 14 mOsm/L raffinose solution prepared in the same HEPES buffer. Disposable cuvettes of 1 × 1 cm were used. After 300 s of equilibration time, 3 measurements were carried out at 25 °C with 15 repetitions each. The average vesicle diameter was in the 80–100 nm range. During the whole preparation procedure, the light exposure of the lipid films and solutions was minimized by using amber glass vials and aluminum foil.

### 3.4. Liposome Swelling Assay

Relative OmpF permeability to a set of 30 compounds from different classes was measured (Figure S1 and Table S1), including both antibiotics and non-antibiotics, as previously described [20,23,26,28]. Substrate solutions were prepared in the same HEPES buffer used for vesicle preparation and checked with the osmometer. An aliquot of 60 μL of the vesicle stock solution was pipetted into 1 mL of the substrate solution and the OD reading was started as fast as possible (dead time < 3 s). Disposable semi-micro cuvettes were used with an optical path length of 1 cm. OD kinetics were recorded for 300 s with a Cary 5000 UV-Vis-NIR spectrometer (Agilent, Santa Clara, CA, USA), at 500 nm, with a bandwidth of 2 nm and 10 pt/s rate. The substrate solution reading before vesicle addition was set as the zero OD. The swelling rate, in OD/min, was obtained from the least-square linear curve fitting of the first 20 s and averaging 4–6 independent measurements. The swelling rate in proteoliposomes was corrected for with the swelling rate possibly observed in control liposomes. Then, the relative permeability was calculated as the ratio between the corrected swelling rate of the substrate and that obtained for glycine, which was set to 100%. In the case of negatively charged species, they were provided (or supplemented) with Na^+^ counterions. In fact, each substrate translocation event must be accompanied by the translocation of the proper number of counterions to keep the transmembrane potential to zero and avoid the apparent impermeability of the porin [28]. For this reason, the observed swelling rates were divided by 2, 3, or 4, in the case of mono-, di-, or trivalent anions, respectively.

### 3.5. Bacterial Cultures and Antimicrobial Activity Test

The *Escherichia coli* BL21 was selected as the biological model for this study as this strain is widely used for MIC determination, providing consistent results due to its stable genetic background and predictable response to antibiotics. In particular, we used the BL21(DE3) *omp8*, characterized by ΔlamB *ompF*::Tn5Δ*ompA*Δ*ompC* [33], and expressing OmpF and OmpC from the K12 strain by the insertion of pGOmpF and pGOmpC plasmids. Notably, such an engineered strain was the same one used for the production, isolation, and purification of the OmpF reconstituted in the proteoliposomes (vide supra). *E. coli* was cultured in Lennox Broth (LB) purchased from Condalab Italia and the stock culture was stored at −80 °C in LB supplemented with 20% glycerol. In order to minimize the variation in experimental conditions and ensure reproducible experimental data, a growth curve test was preliminarily performed and the standard inoculum determined (5 × 10^7^ CFU/mL). During the experiments, bacteria were incubated at 37 °C with shaking (200 rpm) for 24 h. Drugs were primarily dissolved in DMSO to create a stock solution at 5120 μg/mL and stored at −80 °C until use. The MIC was assessed for a set of 38 antibiotics from different classes (Figure S2 and Table S2) by the micro-dilution method in 96-well plates (Thermo Fisher Scientific, Norristown, OA, USA) as previously reported. Briefly, each well was filled with 200 µL of standardized microbial suspension and 5% (*v*/*v*) of different drug concentrations obtained by two-fold serial dilutions in DMSO, reaching a concentration range suitable to infer the MIC value—which is drug-dependent—in line with other studies and as per EUCAST for *E. coli* [Testing TECoAS]. Each experiment was repeated at least 3 times and technical duplicates were present within each plate. Vehicle-treated bacteria represent the control group. The cultures were incubated at 37 °C for 24 h and the turbidimetry was measured by optical density at 600 nm with the microplate reader Tecan Infinite 200 (Tecan Group, Männedorf, Switzerland). The MIC value, expressed in µM, represents the lowest concentration of the drug that inhibits more than 80% compared to the control group growth, and its optical density measure does not significantly differ from that of the original inoculum (baseline at 0 h), as calculated by one-way ANOVA followed by Fisher’s LSD test, setting the significance at *p* < 0.01, performed using Prism 9 (GraphPad Software, San Diego, CA, USA).

### 3.6. Cheminformatic Tools

MarvinSketch v21.20 (Chemaxon, Budapest, Hungary) was used to calculate molecular descriptors that are believed to be important for porin permeation; namely, the acid/base protonation constants (pKas), net charge at pH 7.4, molecular weight, number of rotatable bonds, fused rings and ring counts, logP at pH 7.4, Van der Waals volume and area, polar surface area, minimum and maximum projection area, and dipole moment (details of all the calculation plugins can be found in the User’s Guide at https://docs.chemaxon.com/display/lts-europium/calculations-plugins-in-marvinsketch.md, accessed on 6 March 2025). The pKas were calculated considering a temperature of 298 K and taking possible tautomerization/resonance into account. The logP was calculated by using the ChemAxon model and considering an electrolyte concentration equal to the experimental buffer osmolarity in the liposome swelling assays. All the size-related descriptors were computed by performing a default preliminary search for the minimum energy conformer of the main protonation state at pH 7.4. The same was done for the electric dipole moment calculation.

### 3.7. Machine Learning

We have created a customized ML approach: a combination of classification and regression, where the latter was achieved by decoding the predicted classification values. The classification itself was performed by employing multiple methods, including Logistic Regression (LR) and XGBoost (XGB). Each of the methods was trained with and without regularization (L1, L2, and Elastic Net), where LR used a liblinear solver for all models but the one regularized by Elastic Net, which used a saga solver. The regularization coefficients were set to 1 in both L1 and L2, whereas Elastic Net was controlled by a coefficient ratio set to 0.5. The same regularization settings were used for the XGB models, for which the default training parameters were used (max_dept = 6 and learning_rate = 0.3).

The strategy of comparing LR and XGB was motivated by the different interpretability levels of these models. Keeping simpler but more interpretable models, such as Logistic Regression, allows for deeper insights about the molecular descriptors most relevant to the underlying process [35,36]. A rigorous validation process was implemented, employing stratified K-fold cross-validation with 11 (limited to the minimum number of class members) random train–test splits [37]. This approach ensures the model’s robustness and generalizability by exposing it to multiple training/testing processes, reducing the possibility of overfitting. To comprehensively evaluate the classification models’ performance, multiple metrics were used; namely, the accuracy (measuring the overall correctness of the predictions), precision (assessing the proportion of true positive predictions), recall (evaluating the model’s ability to identify all positive instances), and the Area Under the Receiver Operating Characteristic Curve (ROC-AUC; providing the model’s discriminative ability). The classification results were decoded by using a weighting function that leverages the probabilities of a molecule being assigned to each class (see the supporting material). Once the classification results were decoded, we used the RMSE of the predicted RPC to identify the optimum model (Table 1). For data analysis, visualization, and machine learning tasks, the Python programming language was used. Specifically, the Scikit-learn package [38] was employed. Additional details can be found in [25] and in the references quoted therein. Based on the results obtained in the previously quoted reference, and on the seminal works cited in the Introduction, we limited the descriptors to the ones obtained by Marvin (vide supra) and the statistical descriptors extracted from MD simulations (vide infra). The latter included the electric charge, the minor, semi-major, and major axis of the minimal molecule-containing ellipsoid [39], and the total, transversal, and parallel dipole moment. Definitions, mathematical formulas, and references can be found in the Supplementary Material file. All of these descriptors (excluding the net charge) were not static quantities, but they were monitored along MD trajectories to obtain proper information about their variability. Their statistical distribution was characterized through the minimum set of parameters as indicated by Pearson [40]; namely, the mean value, standard deviation, skewness, and kurtosis, bringing the maximum number of descriptors in our ML models to 39.

### 3.8. Molecular Dynamics Simulations

The molecular dynamics (MD) simulations were prepared and carried out by using an automated procedure that starts with generating the initial structure from the SMILES format [41]. RDKit (Open-source cheminformatics, Release_2024.09.6 https://www.rdkit.org, accessed on 6 March 2025) was used for structure generation and minimization with the UFF force field [42]. The minimized structure was then parameterized with AmberTools23 [43] with Antechamber [44] (University of California, San Francisco, Dept. Pharmaceutical Chemistry, USA) and the GAFF force field [45]. The molecule was placed in a cubic 4 × 4 × 4 nm water box with 0.15 M NaCl (plus counterions if needed) and simulated with Gromacs version 2022.4 (https://www.gromacs.org/, accessed on 6 March 2025). The TIP3P model was used for water molecules [46]. After energy minimization and equilibration, a 300 ns production simulation was run at 300 K in the NPT ensemble by using the Parrinello−Rahman algorithm [47]. Long-range electrostatics were treated with a particle mesh Ewald scheme, while the Coulomb and Lennard-Jones potentials described short-range interactions within a 1 nm cutoff.

## 4. Conclusions

Looking for simplistic correlations with static descriptors, aiming at finding some golden rules for antibiotic design, appears overoptimistic. Aware of the limited size of the used data sets, the presented results show the feasibility of the approach nevertheless, with a potential great impact on drug discovery and development. Of course, the more data fed into the pipeline, the greater the accuracy and the wider the applicability. Furthermore, other experimental data need to be possibly included in the ML modeling to get a more comprehensive prediction of the full complexity of antibacterial activity, such as the affinity for the isolated target, localization of the target inside the bacterial cell, permeability of the lipid bilayer, and susceptibility to efflux systems and to degradative enzymes, in order to dissect the role of these different but concomitant processes towards a more rational drug design. The development of a robust predictive tool that can not only estimate permeability but also anticipate the resulting antibacterial activity would be extremely beneficial to researchers in both academia and industry: it would be a valuable in silico resource for rapidly evaluating a large number of compounds, thereby accelerating the identification of new antibiotic candidates and reducing the associated costs. In the long term, by building separate reliable ML models for permeation, accumulation, and antibacterial activity, it might be possible to dissect the relative weight of permeation and efflux on the latter, gaining the fundamental information necessary to discover scaffolds for designing new effective antibiotics and also to optimize many already tested candidates that failed along the pipeline because of OM permeability issues.

## Figures and Tables

**Table 1:** ML classification models and their accuracy.

Method	Model ID	Accuracy	RMSE (%_RPC_)
Logistic Regression (L2)	LR-I	0.81	28.6
Logistic Regression (L1)	LR-II	0.78	28.0
Logistic Regression (ElasticNet)	LR-III	0.86	22.6
Logistic Regression (No Regularization)	LR-IV	0.81	28.6
XGBoost (Standard)	XG-I	0.78	37.6
XGBoost (L2 Regularization)	XG-II	0.78	37.6
XGBoost (L1 Regularization)	XG-III	0.73	32.6
XGBoost (ElasticNet—L1 and L2)	XG-IV	0.73	34.5

**Table 2:** Results of the linear fitting of the MIC vs. RPC data points.

Model	Model ID	Slope	Intercept
Logistic Regression (L2)	LR**-**I	−0.071	4.265
Logistic Regression (L1)	LR**-**II	−0.068	4.152
Logistic Regression (ElasticNet)	LR**-**III	−0.074	4.041
Logistic Regression (No Regularization)	LR**-**IV	−0.071	4.265
XGBoost (Standard)	XG**-**I	−0.075	4.021
XGBoost (L2 Regularization)	XG**-**II	−0.075	4.021
XGBoost (L1 Regularization)	XG**-**III	−0.085	4.353
XGBoost (ElasticNet—L1 and L2)	XG**-**IV	−0.079	4.117

## Data Availability

The data presented in this study are available on request from the corresponding authors due to reasonable request.

## Supplementary Materials

The following supporting information can be downloaded at: https://www.mdpi.com/article/10.3390/molecules30061224/s1. Definitions and description of ML models and metrices. Figure S1: Structure formula of the substrates used for the relative permeability investigation through the OmpF porin. Table S1: The values obtained with MarvinSketch for selected chemico-physical descriptors of the minimum energy conformer, of the main protonation state at pH 7.4, of the compounds shown in Figure S1. Figure S2: Structure formula of the antibiotics investigated through the micro-dilution method to determine the minimum inhibitory concentration against *E. coli*. Table S2: The values obtained with MarvinSketch for selected chemico-physical descriptors of the minimum energy conformer, of the main protonation state at pH 7.4, of the compounds shown in Figure S2. Table S3: Correlation between the selected chemico-physical descriptors and the relative permeability coefficients (Figure 3). Figure S3: Probability distribution of the descriptors listed in Tables S1 and S3 for the substrates reported in Figure S1. Figure S4: Probability distribution of the interesting statistical descriptors listed in Tables S3 and S4 for the substrates reported in Figure S1. Table S4: Features used in training with their respective origin. Table S5: The top 10 important features in LR-III model.
