# Supplementary material for: Seeking Correlation Among Porin Permeabilities and Minimum Inhibitory Concentrations Through Machine Learning: A Promising Route to the Essential Molecular Descriptors
Source: Molecules. 2025 Mar 9;30(6):1224. doi: 10.3390/molecules30061224 (PMC11944608; doi:10.3390/molecules30061224)
Supplement: Supplementary file 1 [file molecules-30-01224-s001.zip › molecules-3481448-supplementary.pdf]

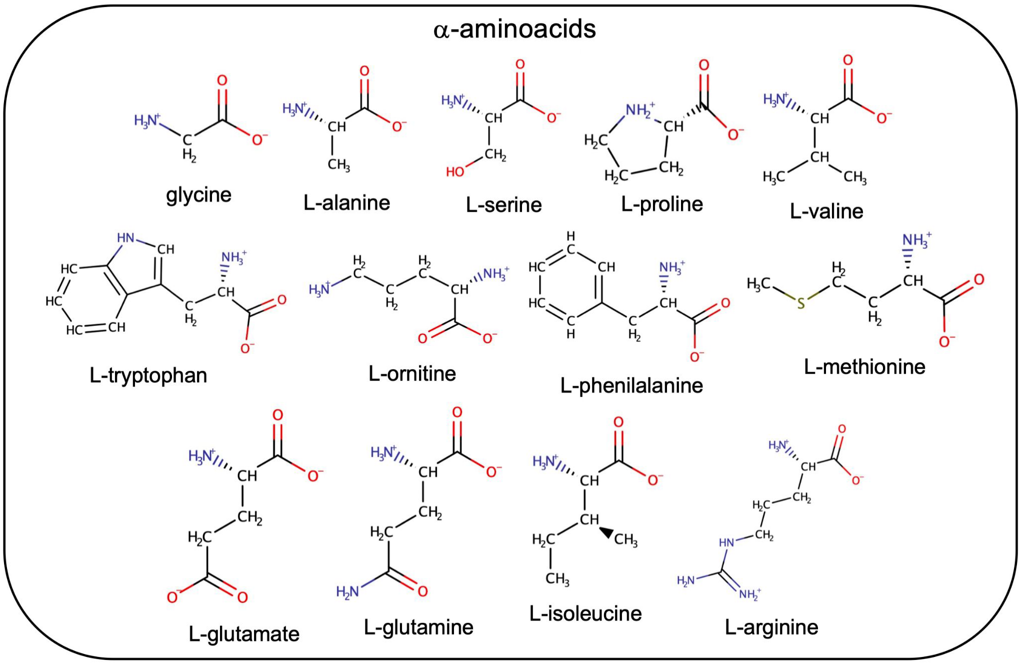

Figure S1 (continue)

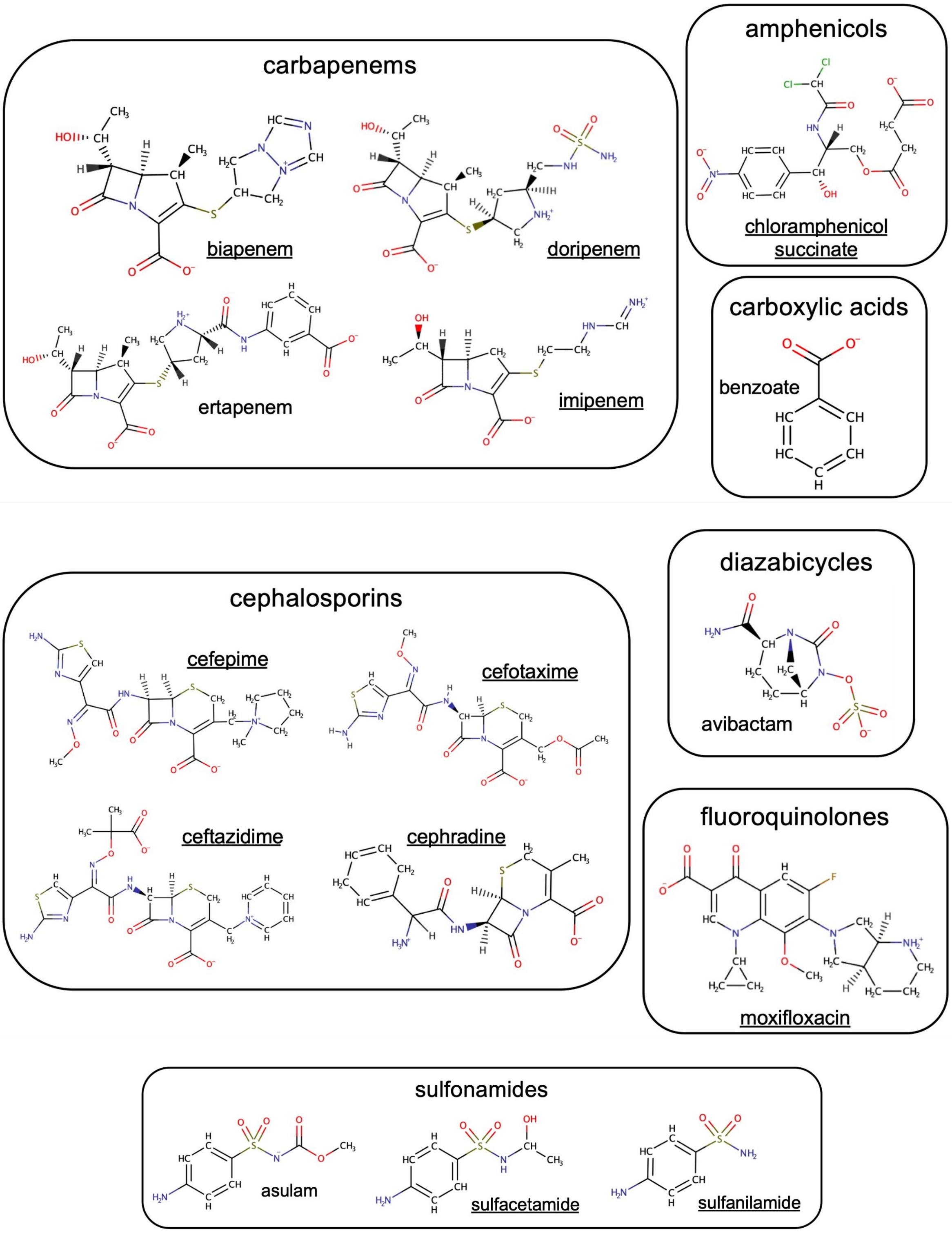

Figure S1 (continue)

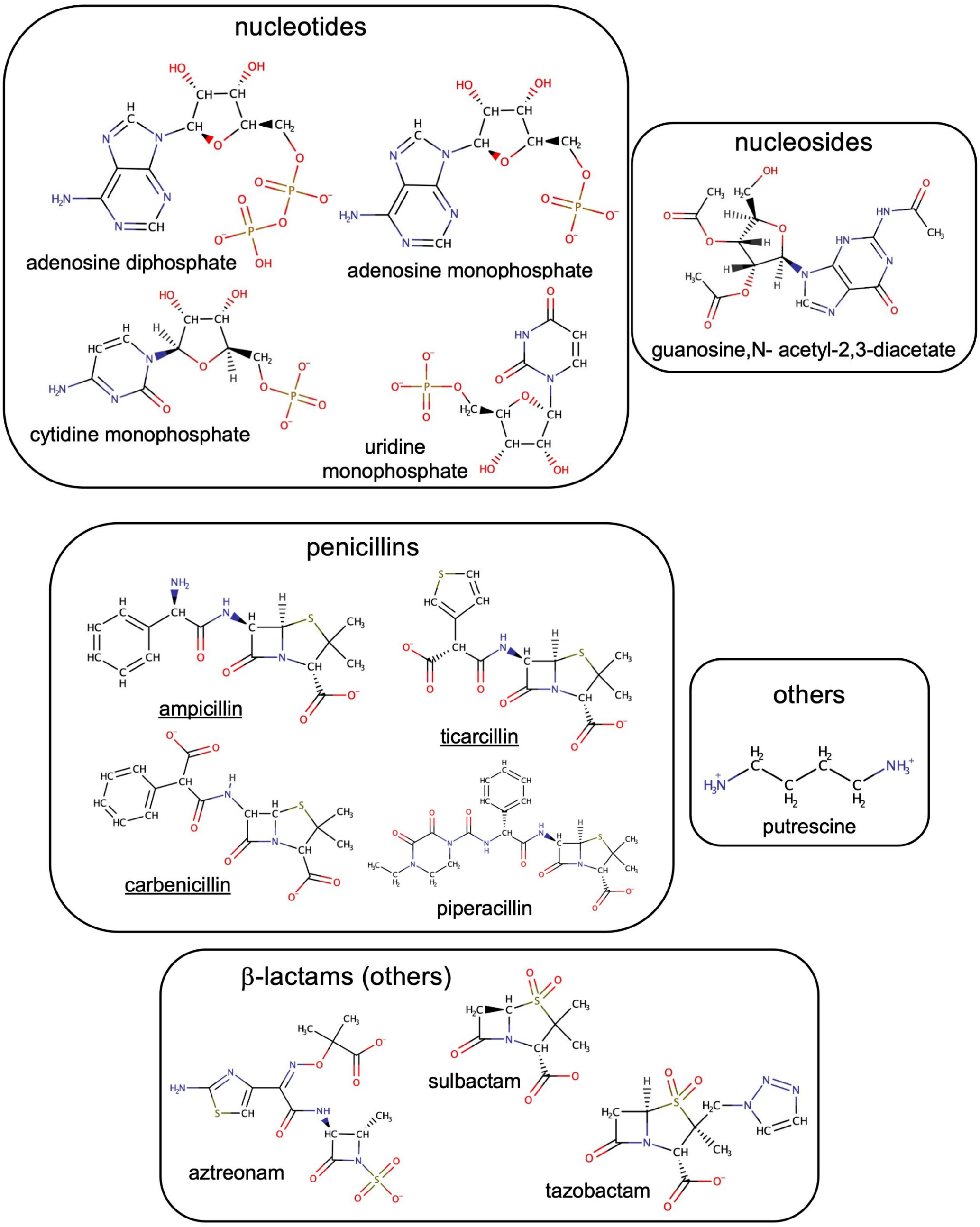

Figure S1. Structure formula of the substrates used for the relative permeability investigation through the OmpF porin. The main protonation state at pH 7.4 is shown, as it was estimated with the MarvinSketch pKa calculation plugin (https://docs.chemaxon.com/display/docs/calculators_pka-plugin.md). Different molecular classes were investigated, namely, α-aminoacids, carbapenems, amphenicols, carboxylic acids, cephalosporins, diazabicycles, fluoroquinolones, sulfonamides, nucleotides, nucleosides, penicillins, other β-lactams, and others. The names of the molecules for which MIC was also measured are underlined.

Table S1. The values obtained with MarvinSketch for selected chemico-physical descriptors of the minimum energy conformer, of the main protonation state at pH 7.4, of the compounds shown in Figure S1.

Name	MW[a] (g/mol)	V_{Vdw}[b] (Å^3)	A_{Vdw}[c] (Å^2)	PSA[d] (Å^2)	MinPA[e] (Å^2)	MaxPA[f] (Å^2)
adenosine diphosphate	425	301	473	258	53	97
adenosine monophosphate	345	256	401	202	46	85
ampicillin	349	299	460	142	54	82
asulam	229	183	301	104	32	63
avibactam	264	199	325	141	34	60
aztreonam	433	331	543	244	57	100
benzoate	121	107	171	40	19	44
biapenem	359	288	468	128	48	93
carbenicillin	376	310	496	155	54	86
cefepime	481	396	639	204	74	106
cefotaxime	454	351	559	230	62	106
ceftazidime	546	435	691	248	77	116
cephradine	349	295	467	142	52	83
chloramphenicol succinate	422	328	524	159	67	105
cytidine monophosphate	321	241	380	191	44	80
doripenem	421	348	566	203	53	110
ertapenem	475	398	606	192	69	120
fosfomycin	137	102	176	83	24	36
glycine	75	68	120	68	19	25
guanosine,N-acetyl-2,3-diacetate	409	333	550	170	59	103
imipenem	299	252	415	144	38	85
L-alanine	89	85	148	68	24	28
L-arginine	175	165	274	131	25	60

L-glutamate	147	127	209	108	25	46
L-glutamine	146	132	218	111	26	48
L-isoleucine	117	119	239	68	27	39
L-methionine	149	138	233	93	25	46
L-ornithine	133	133	225	95	25	48
L-phenylalanine	165	156	253	68	29	54
L-proline	115	108	186	57	28	36
L-serine	105	94	158	88	22	35
L-tryptophan	204	182	292	84	32	62
L-valine	117	119	208	68	26	40
moxifloxacin	401	348	558	90	50	108
piperacillin	517	435	687	185	80	120
putrescine	90	108	193	55	20	39
sulbactam	232	183	310	103	42	52
sulfacetamide	216	184	299	101	40	58
sulfanilamide	172	141	226	95	26	53
tazobactam	299	229	378	134	46	67
ticarcillin	382	301	475	183	50	86
uridine monophosphate	322	238	375	181	42	80
**Name**	**RB**[g]	**RC**[h]	**FR**[i]	**NC**[j] **(/e C)**	**EDM**[k] **(D)**	**logP**[l]
adenosine diphosphate	6	3	2	-2.5	34.4	-7.8
adenosine monophosphate	4	3	2	-1.9	40.9	-6.1
ampicillin	4	3	2	0.0	34.9	-5.2
asulam	2	1	0	-1.0	18.5	-2.6
avibactam	3	2	2	-1.0	19.4	-4.0
aztreonam	6	2	0	-2.0	26.1	-6.8
benzoate	1	1	0	-1.0	14.1	-1.8
biapenem	4	4	4	0.0	24.2	-12.4

carbenicillin	5	3	2	-2.0	12.1	-8.1
cefepime	7	4	2	0.0	25.7	-11.5
cefotaxime	8	3	2	-1.0	19.9	-5.9
ceftazidime	9	4	2	-1.0	53.5	-14.9
cephradine	4	3	2	-0.4	36.3	-5.7
chloramphenicol succinate	11	1	0	-1.0	32.9	-3.0
cytidine monophosphate	4	2	0	-1.9	39.2	-5.8
doripenem	6	3	2	0.0	31.2	-9.3
ertapenem	7	4	2	-1.0	23.0	-9.0
fosfomycin	2	1	0	-1.3	6.8	-3.5
glycine	1	0	0	0.0	13.8	-6.0
guanosine,N-acetyl-2,3-diacetate	7	3	2	0.0	8.3	-1.5
imipenem	6	2	2	0.0	36.8	-11.2
L-alanine	1	0	0	0.0	14.0	-5.6
L-arginine	5	0	0	1.0	30.3	-5.8
L-glutamate	4	0	0	-1.0	15.5	-8.2
L-glutamine	4	0	0	0.0	11.1	-5.9
L-isoleucine	2	0	0	0.0	13.8	-4.6
L-methionine	4	0	0	0.0	14.7	-5.0
L-ornithine	4	0	0	1.0	30.3	-7.2
L-phenylalanine	3	1	0	0.0	13.9	-3.9
L-proline	1	1	0	0.0	14.6	-6.1
L-serine	2	0	0	0.0	12.7	-6.6
L-tryptophan	3	2	2	0.0	13.2	-3.6
L-valine	2	0	0	0.0	13.9	-4.6
moxifloxacin	4	5	4	0.0	66.2	-1.7
piperacillin	6	4	2	-1.0	25.4	-4.0
putrescine	3	0	0	2.0	0.0	-6.3

sulbactam	1	2	2	-1.0	12.2	-5.7
sulfacetamide	2	1	0	0.0	15.5	-0.2
sulfanilamide	1	1	0	0.0	13.1	-0.1
tazobactam	3	3	2	-1.0	12.0	-6.8
ticarcillin	5	3	2	-2.0	19.7	-7.9
uridine monophosphate	4	2	0	-1.9	31.9	-5.9

[a]Molecular Weight; [b]Van der Waals Volume; [c]Van der Waals Area; [d]Polar Surface Area; [e]Minimal Projection Area; [f]Maximal Projection Area; [g]Rototable Bonds; [h]Ring Count; [i]Fused Rings; [j]Net Charge expressed as multiple of the elementary charge; [k]Electric Dipole Moment; [l]logP at pH 7.4.

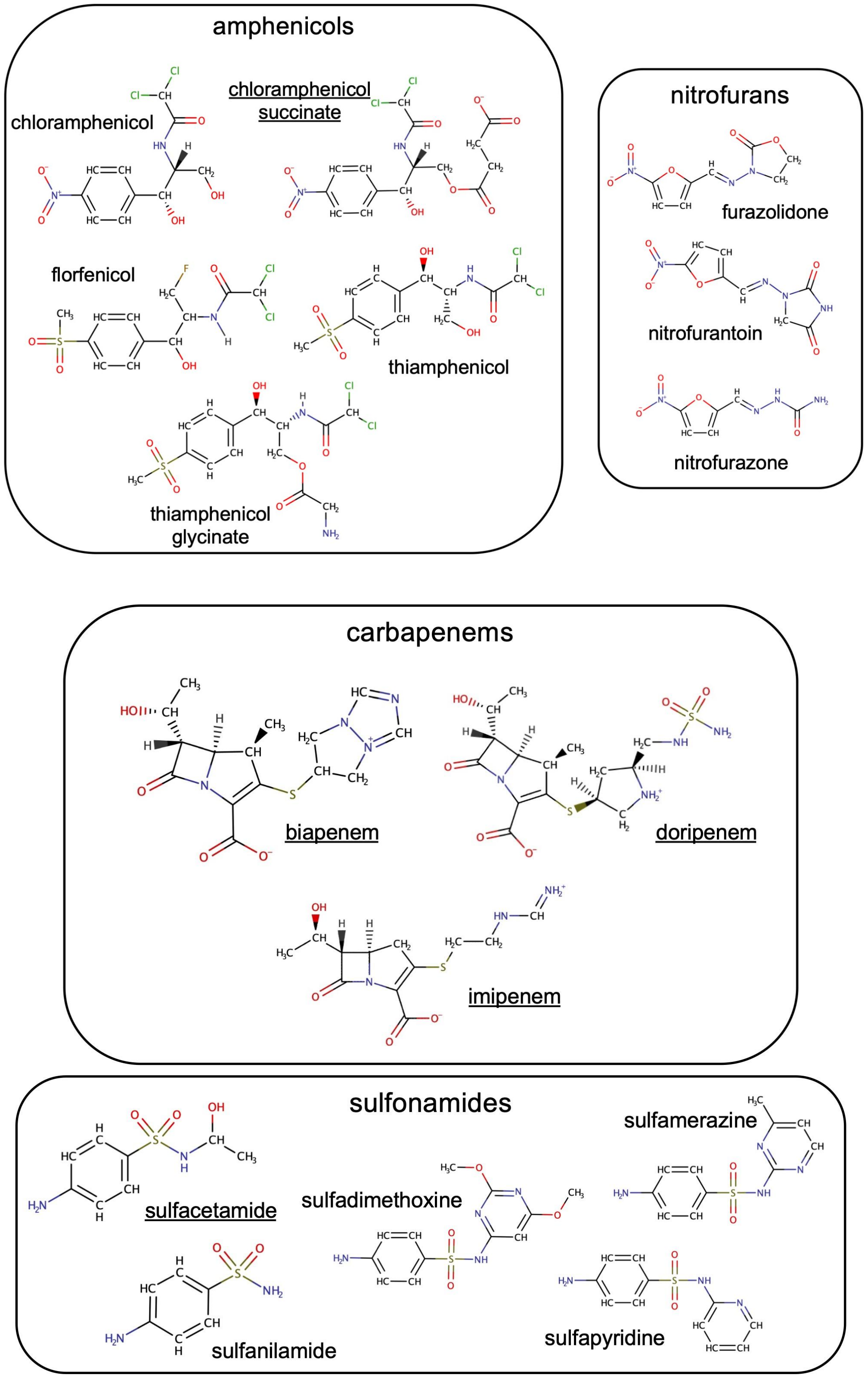

Figure S2 continue

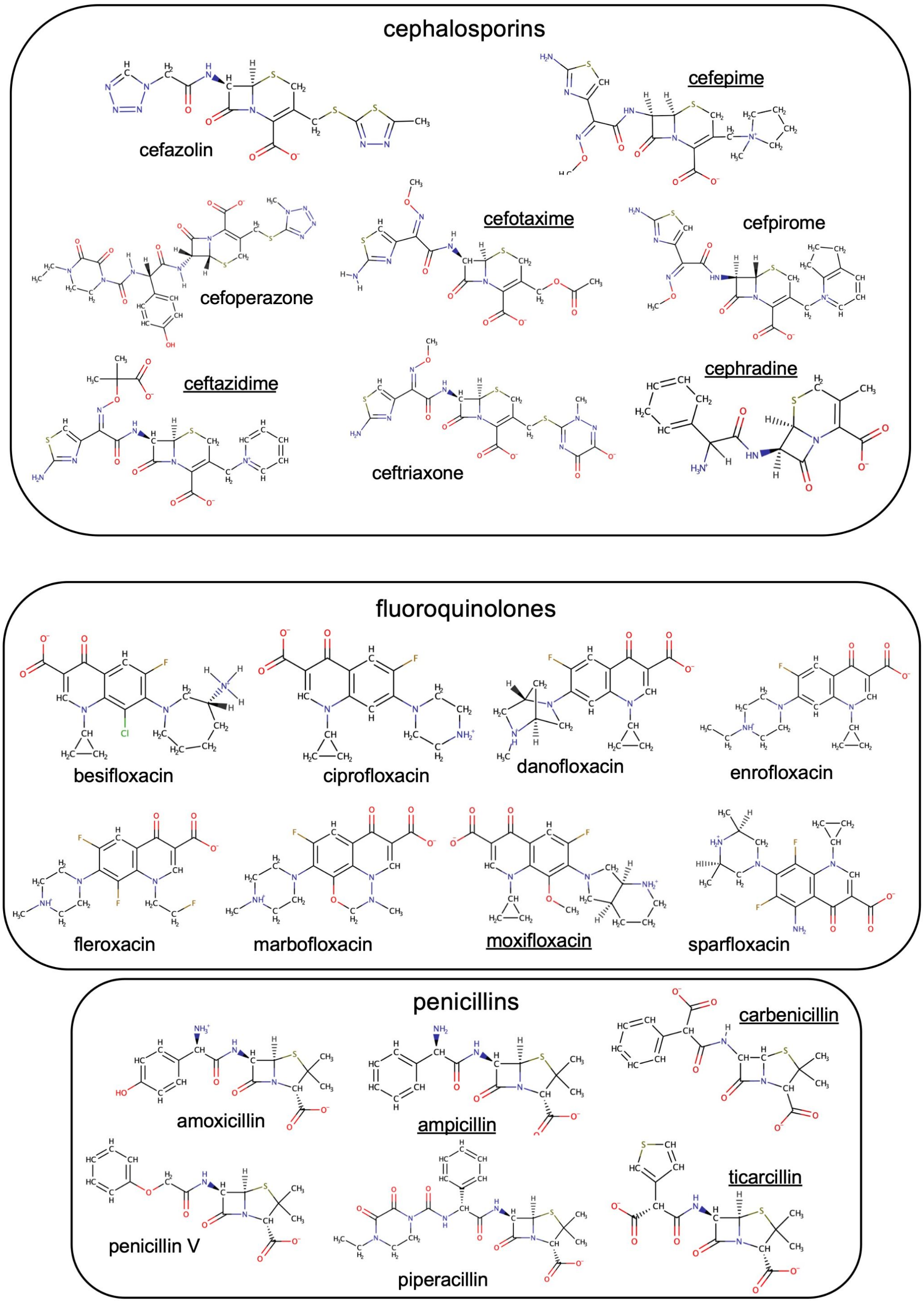

Figure S2. Structure formula of the antibiotics investigated through the micro-dilution method to determine the minimum inhibitory concentration against *E. coli*. The main protonation state at pH 7.4 is shown, as it was estimated with the MarvinSketch pKa calculation plugin (https://docs.chemaxon.com/display/docs/ calculators_pka-plugin.md). Different classes were investigated, namely, amphenicols, nitrofurans, carbapenems, sulfonamides, cephalosporins, fluoroquinolones, and penicillins. The names of the antibiotics for which relative permeability through OmpF was also available are underlined.

**Table S2.** The values obtained with MarvinSketch for selected chemico-physical descriptors of the minimum energy conformer, of the main protonation state at pH 7.4, of the compounds shown in Figure S2.						
**Name**	**MW[a] (g/mol)**	**V_{Vdw}[b] (Å^3)**	**A_{Vdw}[c] (Å^2)**	**PSA[d] (Å^2)**	**MinPA[e] (Å^2)**	**MaxPA[f] (Å^2)**
amoxicillin	362	303	480	160	57	83
ampicillin	349	299	460	142	54	82
besifloxacin	394	331	516	91	56	103
biapenem	359	288	468	128	48	93
carbenicillin	376	310	496	155	54	86
cefazolin	453	337	522	238	61	109
cefepime	481	396	639	204	74	106
cefoperazone	645	513	818	274	96	131
cefotaxime	454	351	559	230	62	106
cefpirome	515	414	656	207	62	130
ceftazidime	546	435	691	248	77	116
ceftriaxone	553	414	648	297	71	118
cephradine	349	295	467	142	52	83
chloramphenicol	323	249	387	113	47	79
chloramphenicol succinate	422	328	524	159	67	105
ciprofloxacin	331	283	449	80	43	97
danofloxacin	346	296	469	66	45	99
doripenem	421	348	566	203	53	110
enrofloxacin	347	306	497	65	46	107
fleroxacin	342	281	452	62	41	97
florfenicol	358	275	435	92	44	85
furazolidone	225	170	280	98	31	60
imipenem	299	252	415	144	38	85
marbofloxacin	355	299	481	78	45	105
moxifloxacin	401	348	558	90	50	108

nitrofurantoin	238	175	287	118	32	60
nitrofurazone	198	150	244	124	29	56
penicillin V	349	293	478	124	49	94
piperacillin	517	435	711	185	80	120
sparfloxacin	392	333	535	106	52	107
sulfacetamide	216	184	299	101	40	58
sulfadimethoxine	310	253	412	123	53	81
sulfamerazine	264	218	349	104	46	69
sulfanilamide	172	141	226	95	26	53
sulfapyridine	248	205	323	91	32	67
thiamphenicol	356	279	439	112	50	83
thiamphenicol glycinate	414	329	525	145	64	100
ticarcillin	382	301	475	183	50	86
**Name**	**RB[g]**	**RC[h]**	**FR[i]**	**NC[j] (/e C)**	**EDM[k] (D)**	**logP[l]**
amoxicillin	4	3	2	-0.6	26.7	-5.5
ampicillin	4	3	2	0.0	34.9	-5.2
besifloxacin	3	4	2	0.0	60.7	-1.0
biapenem	4	4	4	0.0	24.2	-12.4
carbenicillin	5	3	2	-2.0	12.1	-8.1
cefazolin	7	4	2	-1.0	13.8	-7.2
cefepime	7	4	2	0.0	25.7	-11.5
cefoperazone	9	5	2	-1.0	21.2	-5.6
cefotaxime	8	3	2	-1.0	19.9	-5.9
cefpirome	7	5	4	0.0	29.5	-11.2
ceftazidime	9	4	2	-1.0	53.5	-14.9
ceftriaxone	8	4	2	-2.0	39.6	-10.8
cephradine	4	3	2	-0.4	36.3	-5.7
chloramphenicol	6	1	0	0.0	5.7	0.6
chloramphenicol succinate	11	1	0	-1.0	32.9	-3.0

ciprofloxacin	3	4	2	0.0	64.1	-1.2
danofloxacin	3	5	4	-0.4	54.0	0.0
doripenem	6	3	2	0.0	31.2	-9.3
enrofloxacin	4	4	2	-0.4	54.0	0.4
fleroxacin	4	3	2	-0.8	39.3	0.6
florfenicol	6	1	0	0.0	6.6	0.3
furazolidone	3	2	0	0.0	8.3	1.1
imipenem	6	2	2	0.0	36.8	-11.2
marbofloxacin	2	4	3	-0.7	43.6	-0.5
moxifloxacin	4	5	4	0.0	66.2	-1.7
nitrofurantoin	3	2	0	-0.1	8.6	0.0
nitrofurazone	3	1	0	0.0	6.0	0.1
penicillin V	5	3	2	-1.0	23.5	-4.4
piperacillin	6	4	2	-1.0	26.2	-5.2
sparfloxacin	2	4	2	0.0	62.6	-0.1
sulfacetamide	2	1	0	0.0	15.5	-0.2
sulfadimethoxine	4	2	0	-0.8	12.0	0.7
sulfamerazine	2	2	0	-0.7	19.6	0.0
sulfanilamide	1	1	0	0.0	13.1	-0.1
sulfapyridine	2	2	0	-1.0	13.7	0.0
thiamphenicol	6	1	0	0.0	6.2	-0.6
thiamphenicol glycinate	9	1	0	0.5	18.8	-1.4
ticarcillin	5	3	2	-2.0	19.7	-7.9
amoxicillin	4	5	4	0.0	66.2	-1.7
ampicillin	3	2	0	-0.1	8.6	0.0
[a]Molecular Weight; [b]Van der Waals Volume; [c]Van der Waals Area; [d]Polar Surface Area; [e]Minimal Projection Area; [f]Maximal Projection Area; [g]Rototable Bonds; [h]Ring Count; [i]Fused Rings; [j]Net Charge expressed as multiple of the elementary charge; [k]Electric Dipole Moment; [l]logP at pH 7.4.						

Table S3. Correlation between the selected chemico-physical descriptors and the relative permeability coefficients (Figure 3).

**MW**[a]	**V_{VdW}**[b]	**A_{VdW}**[c]	**PSA**[d]	**MinPA**[e]	**MaxPA**[f]	**RB**[g]	**RC**[h]	**FR**[i]	**NC**[j]	**EDM**[k]	**logP**[l]
-0.65	-0.59	-0.59	-0.57	-0.58	-0.60	-0.37	-0.59	-0.42	+0.61	-0.22	-0.08

[a]Molecular Weight (g/mol); [b]Van der Waals Volume ($Å^3$); [c]Van der Waals Area ($Å^2$); [d]Polar Surface Area ($Å^2$); [e]Minimal Projection Area ($Å^2$); [f]Maximal Projection Area ($Å^2$); [g]Rototable Bonds; [h]Ring Count; [i]Fused Rings; [j]Net Charge expressed as multiple of the elementary charge; [k]Electric Dipole Moment (D); [l]logP at pH 7.4.

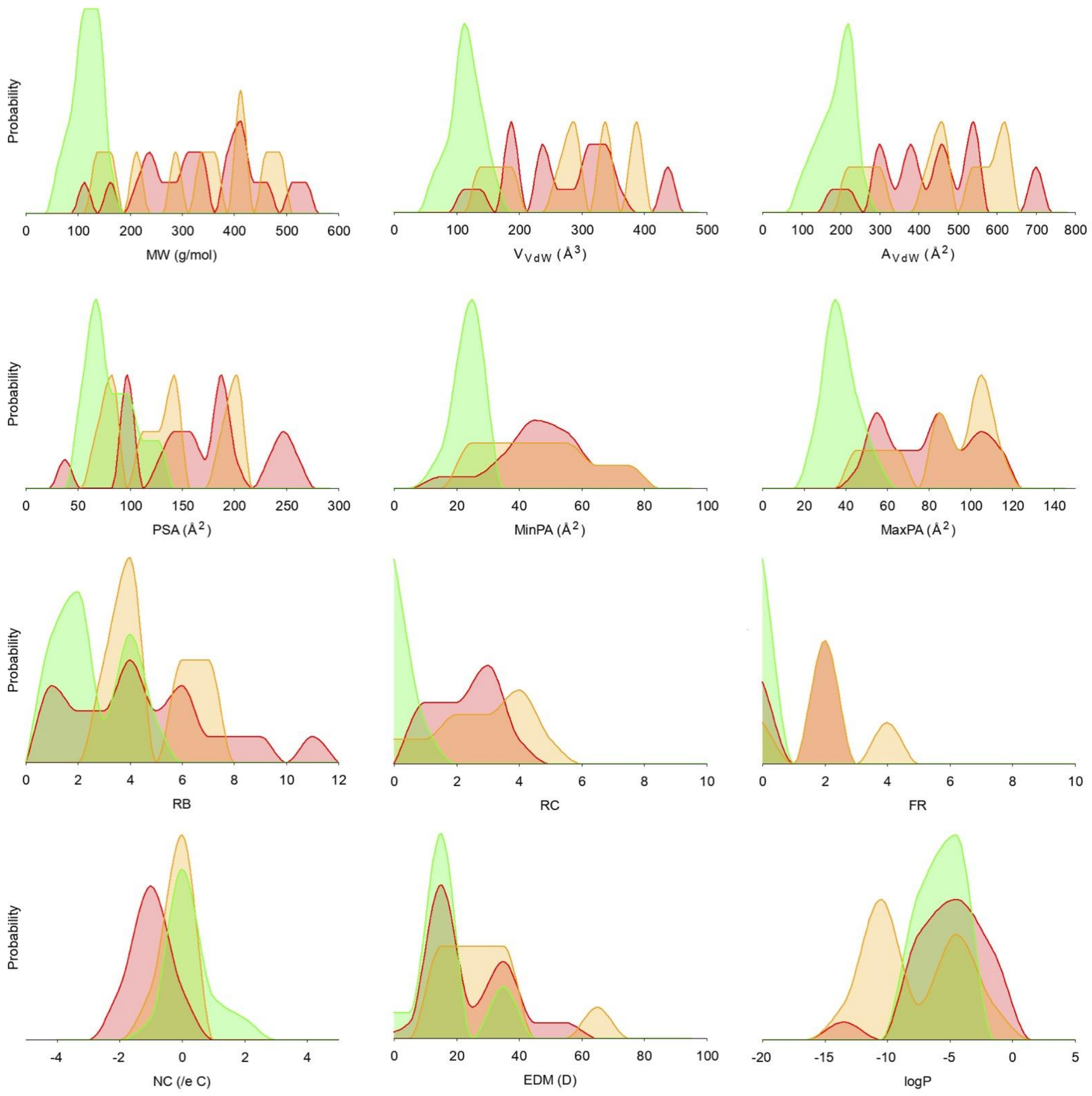

Figure S3. Probability distribution of the descriptors listed in Table S1 and Table S3 for the substrates reported in Figure S1, divided arbitrarily into good (green, RPC > 60), intermediate (orange, 60 > RPC > 30), and bad (red, RPC < 30) permeators according to Figure 3. MW (Molecular Weight); V_{VdW} (Van der Waals Volume); A_{VdW} (Van der Waals Area); PSA (Polar Surface Area); MinPA (Minimal Projection Area); MaxPA (Maximal Projection Area); RB (Rotatable Bonds); RC (Ring Count); FR (Fused Rings); NC (Net Charge); EDM (Electric Dipole Moment); logP (decimal logarithm of the octanol/water partition coefficient at pH 7.4).

ML modeling: Logistic Regression

Logistic regression is a statistical method used for binary classification tasks. It models the probability that a given input belongs to a particular class. The logistic regression model predicts the probability $P(Y=1|X)$ that the dependent variable Y is equal to 1 given the independent variables X. This probability is modeled using the logistic function, also known as the sigmoid function, defined as:

$$P(Y=1|X) = \frac{1}{1+e^{-z}} \quad (1)$$

where z is the linear combination of the independent variables and their corresponding coefficients:

$$z = \beta_0 + \beta_1 X_1 + \beta_2 X_2 + \ldots + \beta_n X_n \quad (2)$$

Here, $(\beta_0, \beta_1, \ldots, \beta_n)$ are the coefficients (or weights) associated with each independent variable $(X_1, X_2, \ldots, X_n)$ respectively. The logistic function constrains the predicted probabilities to lie between 0 and 1, making it suitable for binary classification tasks. The decision boundary is typically set at 0.5, where probabilities greater than 0.5 are classified as belonging to one class, and probabilities less than 0.5 are classified as belonging to the other class. Logistic regression is trained using maximum likelihood estimation to find the optimal values of the coefficients that best fit the data.

ML modelling: XGBoost

XGBoost (eXtreme Gradient Boosting) is a machine learning algorithm based on gradient boosting, designed for high performance in classification and regression tasks. It makes an ensemble of decision trees consecutively, with each new tree correcting the errors of the previous ones. Predictions are the sum of all tree outputs:

$$\hat{y} = \sum_{k=1}^{K} f_k(x) \quad (3)$$

Here, K is the total number of trees, and f_k is the k-th tree. XGBoost minimizes an objective function combining a loss term L (e.g., log loss for classification) and a regularization term Ω to control model complexity:

$$\text{Objective} = \sum_{i=1}^{n} L(y_i, \hat{y}_i) + \sum_{k=1}^{K} \Omega(f_k) \quad (4)$$

XGBoost includes key features like L_1 and L_2 regularization, automatic handling of missing values, efficient tree pruning, and parallel processing for fast training. For classification, outputs can be transformed into probabilities using sigmoid (binary) or softmax (multi-class). Hyperparameters like learning rate and tree depth allow fine-tuning for optimal performance.

ML metrics

An important point in judging the quality of a proposed ML model is the choice of appropriate metrics. In this work, we have chosen accuracy for assessing the model performance. The standard accuracy is defined as

$$\text{Accuracy}_i = \frac{\text{TP}_i + \text{TN}_i}{\text{TP}_i + \text{TN}_i + \text{FP}_i + \text{FN}_i} \quad (5)$$

where, T and F are used for "true" and "false", and P and N for "positive" and "negative". Accuracy measures how often our model makes correct predictions, regardless of the class. Since here, we are dealing with three classes, we have to use an advanced version of accuracy which is called weighted average accuracy:

$$\text{Weighted Accuracy} = \frac{\sum_{i=1}^{n} \text{Accuracy}_i \cdot \text{Support}_i}{\sum_{i=1}^{n} \text{Support}_i} \quad (6)$$

where *Support* is the total number of instances in class *i*.

Decoding function.

To decode the predicted classification values, we used the probabilities obtained by predicting classes with both the classification models listed previously. Namely, the idea is to limit the maximum relative permeability coefficient to 150 and to spread the permeability values between 0 and 150 by means of the following algorithm:

```
def calculate_weighted_score(probs):
```

```
    p_neg1 = optimal_a - probs[0] * optimal_a
    p_0 = (optimal_b + optimal_a) / 2 + probs[1] * (optimal_b -
optimal_a) / 2
    p_1 = optimal_b + (probs[2] * (max_p - optimal_b))
    return probs[0] * p_neg1 + probs[1] * p_0 + probs[2] * p_1
```

Here, optimal_a and optimal_b are class boundaries, probs[0:2] are probabilities of each class and max_p is the maximum relative permeability coefficient.

Statistical moments

In this manuscript, we used the terms 'statistical descriptors' and 'statistics' interchangeably. By both, we referred to the statistical moments of a given distribution (molecular descriptor values collected during one MD simulation). The formal definitions of the four statistical moments are here presented:

- **Mean**

  The first moment, defined as the expected value of a distribution x:

  $$\mu = E[x] \quad (7)$$

- **Standard deviation**

  The second moment is the variance, and the standard deviation is its positive square root:

  $$\sigma = \sqrt{E[(x-\mu)^2]} \quad (8).$$

- **Skewness:**

  The third moment, skewness, measures the asymmetry of a distribution (for a normal distribution, it is identically zero):

  $$\gamma = E[((x-\mu)/\sigma)^3] \quad (9).$$

- **Kurtosis:**

  The fourth moment quantifies the proportion of tails in the total body of data. It is always positive because it is proportional to the fourth power:

  $$k = E[((x-\mu)/\sigma)^4] \quad (10).$$

For the sake of simplicity, skewness and kurtosis are given in their standardized forms.

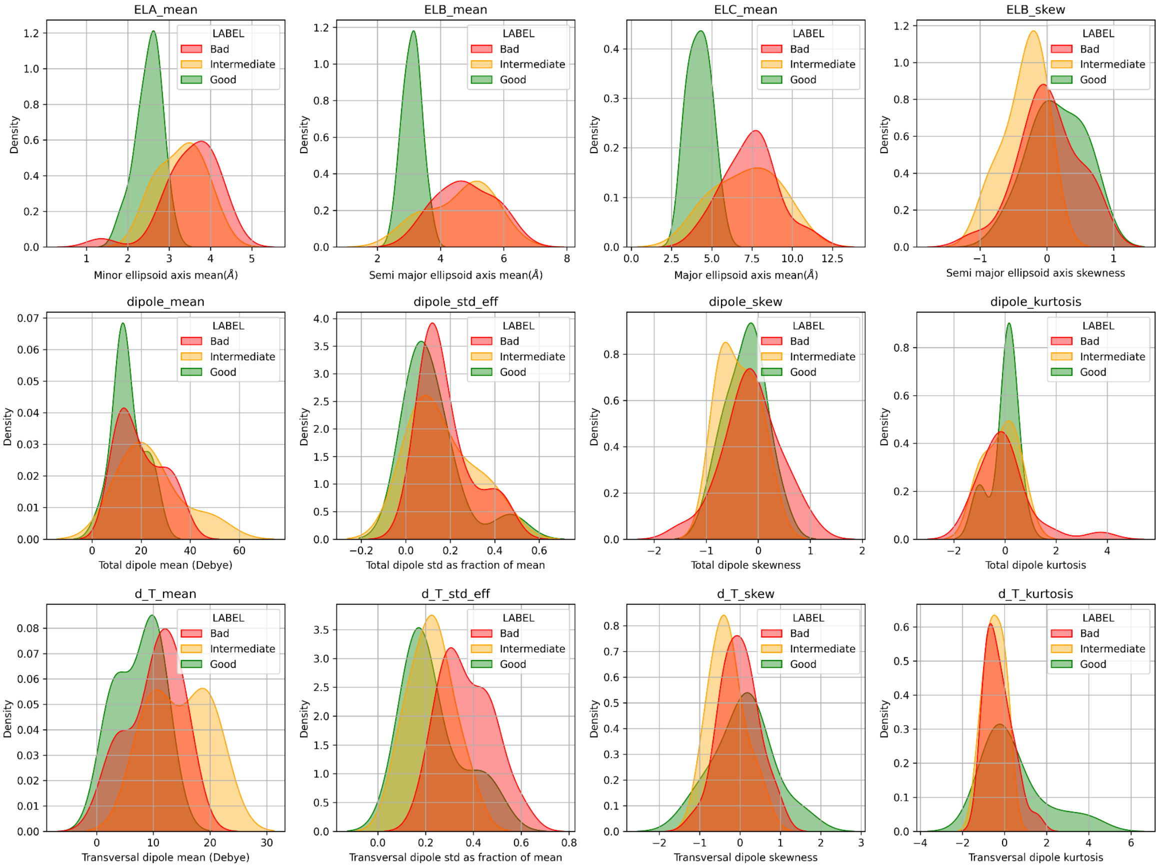

Figure S4. Probability distribution of the interesting statistical descriptors listed in Table S3 and Table S4 for the substrates reported in Figure S1, divided arbitrarily into good (green, RPC > 60), intermediate (orange, 60 > RPC > 30), and bad (red, RPC < 30) permeators according to Figure 3.

Table S4. Features used in training with their respective origin.

**Feature**	**Origin**
Net charge pH = 7.4	MARVIN
Rotable bond	MARVIN
Fused Ring	MARVIN
Ring count	MARVIN
log D	MARVIN
Volume Van der Waals	MARVIN
Min proj area	MARVIN
Max proj area	MARVIN
Dipole Moment (D)	MARVIN
PSA (Polar Surface Area)	MARVIN
Area Van der Waals	MARVIN
PSA/VdW Area	MARVIN
Min Area/VdW Area	MARVIN
Min Area/Max Area	MARVIN
charge_sim	RDKIT
ELA_mean	MD
ELA_std	MD
ELA_skew	MD
ELA_kurt	MD
ELB_mean	MD
ELB_std	MD
ELB_skew	MD
ELB_kurt	MD
ELC_mean	MD
ELC_std	MD
ELC_skew	MD
ELC_kurt	MD
dipole_mean	MD
dipole_std_eff	MD

dipole_skew	MD
dipole_kurtosis	MD
d_P_mean	MD
d_P_std_eff	MD
d_P_skew	MD
d_P_kurtosis	MD
d_T_mean	MD
d_T_std_eff	MD
d_T_skew	MD
d_T_kurtosis	MD

Table S5. The top 10 important features in LR-III model.

Feature	Importance
Net charge pH = 7.4	0.743
log D	0.585
Transversal dipole standard deviation	0.578
Simulation charge	0.515
Transversal dipole kurtosis	0.445
Second ellipsoid diameter skewness	0.392
PSA/VdW Area	0.342
PSA (Polar Surface Area)	0.329
Transversal dipole mean	0.302
Ring count	0.266
